# Effects of host restriction factors and the HTLV-1 subtype on susceptibility to HTLV-1-associated myelopathy/tropical spastic paraparesis

**DOI:** 10.1186/s12977-017-0350-9

**Published:** 2017-04-19

**Authors:** Satoshi Nozuma, Eiji Matsuura, Daisuke Kodama, Yuichi Tashiro, Toshio Matsuzaki, Ryuji Kubota, Shuji Izumo, Hiroshi Takashima

**Affiliations:** 1 Department of Neurology and Geriatrics, Kagoshima University Graduate School of Medical and Dental Sciences, 8-35-1 Sakuragaoka, Kagoshima, 890-8520 Japan; 2 Division of Molecular Pathology, Center for Chronic Viral Diseases, Kagoshima University Graduate School of Medical and Dental Sciences, 8-35-1 Sakuragaoka, Kagoshima, 890-8520 Japan

**Keywords:** HTLV-1, HAM/TSP, Transcontinental subtype, TRIM5α

## Abstract

**Background:**

Although human T-lymphotropic virus type 1 (HTLV-1) infection is a prerequisite for the development of HTLV-1-associated myelopathy/tropical spastic paraparesis (HAM/TSP), specific provirus mutations in HAM/TSP have not yet been reported. In this study, we examined whether HAM/TSP patients had the disease-specific genomic variants of HTLV-1 by analyzing entire sequences of HTLV-1 proviruses in these patients, including familial cases. In addition, we investigated the genetic variants of host restriction factors conferring antiretroviral activity to determine which mutations may be related to resistance or susceptibility to HAM/TSP.

**Results:**

The subjects included 30 patients with familial HAM/TSP (f-HAM/TSP), 92 patients with sporadic HAM/TSP (s-HAM/TSP), and 89 asymptomatic HTLV-1 carriers (ACs). In all 211 samples, 37 samples (18%) were classified into transcontinental subtype and 174 samples (82%) were classified as Japanese subtype. Among three groups, the percentage of transcontinental subtype in f-HAM/TSP, s-HAM/TSP and ACs was 33, 23 and 7%, respectively. The frequency of transcontinental subtype was significantly higher in both f-HAM/TSP (*p* < 0.001) and s-HAM/TSP (*p* < 0.001) than in ACs. Fifty mutations in HTLV-1 sequences were significantly more frequent in HAM/TSP patients than in ACs, however, they were common only in transcontinental subtype. Among these mutations, ten common mutations causing amino acid changes in the HTLV-1 sequences were specific to the transcontinental subtype. We examined host restriction factors, and detected a rare variant in TRIM5α in HAM/TSP patients. The patients with TRIM5α 136Q showed lower proviral loads (PVLs) than those with 136R (354 vs. 654 copies/10^4^ PBMC, *p* = 0.003). The patients with the 304L variant of TRIM5α had significantly higher PVLs than those with 304H (1669 vs. 595 copies/10^4^ PBMC, *p* = 0.025). We could not find any HAM/TSP-specific mutations of host restriction factors.

**Conclusions:**

Transcontinental subtype is susceptible to HAM/TSP, especially in familial cases. Ten common mutations causing amino acid changes in the HTLV-1 gene were specific to the transcontinental subtype. TRIM5α polymorphisms were associated with PVLs, indicating that TRIM5α could be implicated in HTLV-1 replication.

**Electronic supplementary material:**

The online version of this article (doi:10.1186/s12977-017-0350-9) contains supplementary material, which is available to authorized users.

## Background

Human T-lymphotropic virus type 1 (HTLV-1) is the etiologic agent of both adult T cell leukemia (ATL) [1, 2] and an inflammatory neurologic disease, HTLV-1-associated myelopathy/tropical spastic paraparesis (HAM/TSP) [3, 4]. HAM/TSP is clinically characterized by chronic progressive spastic paraparesis, urinary incontinence and mild sensory disturbance. The precise pathophysiological mechanism underlying this disease remains unclear. However, the immune response of the HTLV-1-specific CD8-positive cytotoxic T lymphocytes against HTLV-1-infected CD4-positive lymphocytes migrating into the central nervous systems is a pivotal factor for development of HAM/TSP [5].

HTLV-1 is a complex retrovirus. Its genome contains three typical genes (*gag*, *pol*, and *env*) flanked by long terminal repeat (LTR) sequences at both the 5′ and 3′ ends. In addition, a region known as pX encodes regulatory proteins (Tax, Rex and HBZ) and accessory proteins (p12, p13, and p30) [6]. HTLV-1 is segregated into seven major genetic subtypes (a–g) based on the nucleotide diversity of its LTR region [7]. The cosmopolitan subtype is further divided into five sub-subtypes: (A) Transcontinental, (B) Japanese, (C) West African, (D) North African, and (E) Peruvian Black [8]. While HTLV-1 proviral loads (PVLs) is an important risk factor for HAM/TSP [9], most studies of HTLV-1 genotype have reported no correlation between nucleotide substitutions and the risk of HAM/TSP [10, 11]. The recent analysis of complete HTLV-1 sequence could not show the specific mutation in clinical states from Brazil [11]. Only Furukawa et al. [12] reported the association between the variation of HTLV-1 *tax* gene and the risk of HAM/TSP.

Several host genetic factors, including human leukocyte antigen (HLA) and non-HLA gene polymorphisms affect the occurrence of HAM/TSP [13]. We reported familial clusters of HAM/TSP [14]. These findings indicate that genetic backgrounds may affect individual susceptibility to HAM/TSP. Host restriction factors are important as an initial or early line of defense against infection. In human immunodeficiency virus (HIV) infection, several host restriction factors such as APOBEC3 family, TRIM, tetherin, and SAMHD1 have been well investigated with regard to the biological mechanism of the antiviral response and mutations or polymorphisms of these genes [15, 16]. In HTLV-1 infection, APOBEC3G and SAHMD1 have been studied in terms of antiretroviral activity. APOBEC3G belongs to a family of cytidine deaminases and acts as a potent host restriction factor of retroviral replication through G-to-A hypermutations [17]. In ATL, APOBEC3G generates nonsense mutation in HTLV-1 proviral genomes [18]. SAMHD1 limits HTLV-1 infection of monocyte via STING-Mediated apoptosis [19]. However, no clinical report has been published regarding the genetic variations of host restriction factors in HTLV-1-associated diseases.

DNA sequencing has been remarkably advanced by emerging high-throughput technology. An increased number of disease susceptibility genes in sporadic disease and disease-causing genes in Mendelian diseases have been identified in human genomes [20]. In viral genomes, complete sequencing has been readily obtained and variants have been identified [21, 22].

In this study, we examined whether HAM/TSP patients had the disease-specific genomic variants of HTLV-1 by analyzing entire sequences of proviruses in the patients, including familial cases. In addition, host restriction genes were sequenced to investigate an association with the development of HAM/TSP.

## Methods

### Study population

Peripheral blood samples were obtained from 30 patients with familial HAM/TSP (f-HAM/TSP), 92 patients with sporadic HAM/TSP (s-HAM/TSP), and 89 asymptomatic carriers (ACs) after obtaining informed consent. Peripheral blood mononuclear cells (PBMCs) were separated from the blood by Ficoll gradient centrifugation. The study population was enrolled in our database from 1987 to 2014. Patients with HAM/TSP were diagnosed according to the World Health Organization diagnostic criteria [23]. f-HAM/TSP cases were identified as patients with multiple family members suffering from HAM/TSP [14]; we were able to conduct paired analysis in 18 cases from nine families. This study was approved by the Institutional Review Board of Kagoshima University. All participants provided written informed consent.

### Sequencing the complete provirus genomes

DNA was extracted from PBMCs using the DNeasy Blood and Tissue kit (Qiagen, GmbH, Hilden, Germany) according to the manufacturer’s instructions. HTLV-1 proviral loads (PVLs) in the PBMCs were assayed using quantitative PCR with the ABI PRISM 7700TM sequence detection system as reported previously [9]. The complete provirus genome was amplified by nested PCR of the extracted DNA as reported previously [18]. Initially, we amplified two overlapping fragments of the HTLV-1 gene from 100 ng of genomic DNA. PCR products were then subjected to nested PCR. The final PCR products were purified with the MinElute PCR purification kit (Qiagen, GmbH, Hilden, Germany). All PCR products were ligated with the specified indexes, and simultaneously screened on a MiSeq sequencing system in accordance with the manufacturer’s protocol.

### Data analysis

The raw sequencing reads were analyzed using CLC genomics Workbench v7.5 (CLC Bio, Aarhus, Denmark). The reads were trimmed using the quality score limit of 0.05 and maximum limit of 2 ambiguous nucleotides. Initially, the reads were mapped to the HTLV-1 complete genome (GenBank accession no. AB513134), allowing up to 2 mismatches [24]. All bases of the alignment were evaluated and single nucleotide polymorphisms were called using the Fixed Ploidy Variant Detection tools of the CLC Genomics Workbench. All comparison alignments were performed and phylogenetic tree was constructed by neighbor-joining method with 1000 bootstrap replications using CLC Genomics Workbench [25]. Five complete HTLV-1 sequences, two transcontinental subtypes (Rk13 and BOI) and three Japanese subtypes (ATK, ATL-YS, and AB513134), were used to construct the phylogenetic tree. Next, to analyze the transcontinental subtype sequence, we constructed the consensus sequence from 37 transcontinental subtypes detected in this study. This consensus sequence was designated “transcontinental subtype reference” and was used as the reference to realign the sequence belonging to transcontinental subtypes and identify possible mutations.

### Whole exome sequence

Three micrograms of genomic DNA was processed with the Agilent SureSelect Human All Exon v4 + UTRs capture kit (Agilent, Santa Clara, CA, USA) according to the manufacture’s instructions. The captured DNA was sequenced on an Ilumina Hiseq 2000 (San Diego, CA). Sequences were aligned to the human reference genome (NCBI37/hg19) using the Burrows-Wheeler Aligner (BWA) [26]. Variant calling was performed using SAMtools [27]. Variants were annotated using in-house scripts, which provided the variants list. Previously known variants were annotated from the 1000 Genomes and dbSNP137. We obtained the predicted functional scores of all non-synonymous variants with five prediction algorithms, including SIFT, PolyPhen2, LRT, PhyliP and MutationTaster, using scores obtained from the NSFP database [28]. We obtained mutations or polymorphisms of the candidate gene such as APOBEC3D/F/G/H, TRIM5α, tetherin, and SAMHD1 from whole exome sequence. Rare variants defined minor allele frequency (MAF) ≦1%.

### Statistical analysis

Data were analyzed using SPSS-21 software (SPSS, Chicago, Illinois). Statistical analyses were performed using parametric (*t* test) and non-parametric tests (Mann–Whitney *U* test) for continuous variables and χ^2^ (Pearson χ^2^ test/Fisher’s exact test) for categorical variables. Differences were considered significant when *p* < 0.05. Linkage disequilibrium (LD) was analyzed by Haploview software [29].

### Nucleotide sequence accession numbers

The nucleotide sequences of all transcontinental subtypes used for constructing the consensus sequence in this study were submitted to the DDBJ (DNA Data Bank of Japan) database under accession numbers LC192500–192536.

## Results

### Characteristics of study population

The characteristics of the 211 patients included in this study are summarized in Table 1. We analyzed whole HTLV-1 sequences in 211 samples. The median age of s-HAM/TSP cases was higher than those of f-HAM/TSP and ACs. The percentages of males in the f-HAM/TSP, s-HAM/TSP, and AC groups were 17, 32 and 40%, respectively. Patients with f-HAM/TSP or s-HAM/TSP had significantly higher median PVLs than ACs (Table 1).

**Table 1:** Clinical findings, HTLV-1 subtype, proviral loads, and HTLV-1 sequence changes in patients of HAM/TSP and ACs

	f-HAM/TSP (n = 30)	s-HAM/TSP (n = 92)	ACs (n = 89)	*p* value
Age (years old)	54.7 ± 13.3	58.4 ± 13.2	52.7 ± 14.4	0.021
Male (N, %)	5, 17%	29, 32%	36, 40%	0.052
Transcontinental/Japanese subtype (n)	10/20	21/71	6/83	<0.001
Anti-HTLV-1 antibodies^e^	20,992 ± 33,571 (N = 29)	30,707 ± 43,985 (N = 80)	7667 ± 17,209 (N = 80)	<0.001
HTLV-1 proviral loads (copies/10^4^ PBMCs)	819 ± 743	813 ± 936	366 ± 657	<0.001
No. of SNPs (reference AB513134)^a^	34.2 ± 18.7	29.6 ± 19.2	22.3 ± 12.0	0.004
No. of SNPs (each reference)^b^	21.4 ± 6.8	20.3 ± 7.5	19.5 ± 5.3	0.392
No. of G-to-A mutations (reference AB513134)^c^	10.2 ± 4.6	8.9 ± 4.4	7.4 ± 3.2	0.003
No. of G-to-A mutations (each reference)^d^	6.6 ± 2.5	6.3 ± 2.4	6.7 ± 2.2	0.422

Data are mean ± SD

*f*-*HAM/TSP* familial HTLV-1 associated myelopathy/tropical spastic paraparesis (HAM/TSP) patients, *s*-*HAM/TSP* sporadic HAM/TSP patients, *ACs* asymptomatic HTLV-1 carriers

^a^Number of SNPs of complete HTLV-1 sequences using the data mapped to AB513134

^b^Number of SNPs of complete HTLV-1 sequences using the data mapped to each reference; transcontinental subtype, consensus transcontinental reference; Japanese subtype, AB513134

^c^Number of G-to-A mutations of complete HTLV-1 sequences using the data mapped to AB513134

^d^Number of G-to-A mutations of complete HTLV-1 sequences using the data mapped to each reference; transcontinental subtype, consensus transcontinental reference; Japanese subtype, AB513134

^e^Particle aggregation method

### Characterization of HTLV-1 subtype

**Fig. 1:**
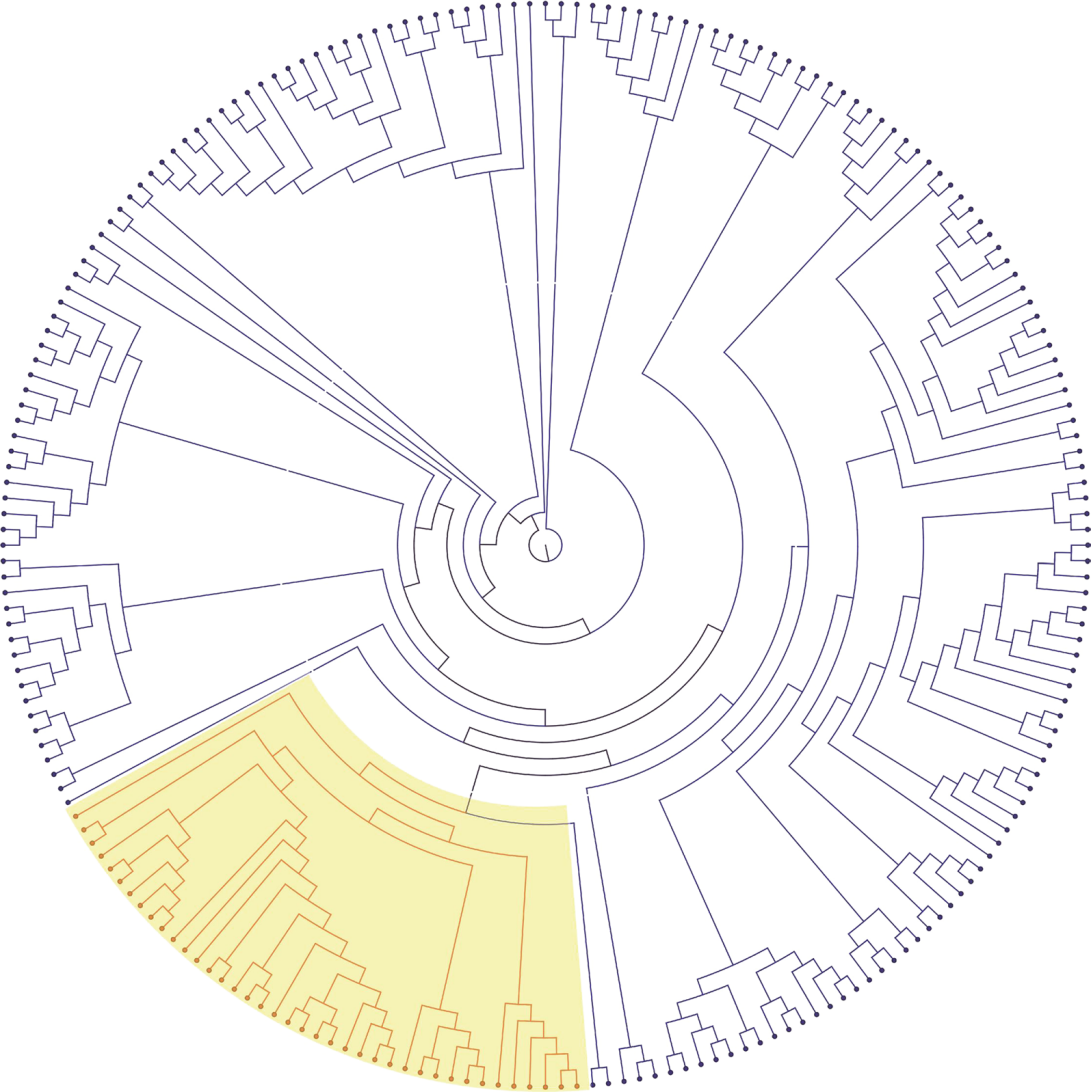
Phylogenetic tree of HTLV-1 strains based on complete sequence of 211 samples. *Colored* (*red*, transcontinental subtype; *blue*, Japanese subtype) branches exhibit samples and reference sequences. Reported complete HTLV-1 sequences, two transcontinental subtypes (Rk13 and BOI) and three Japanese subtypes (ATK, ATL-YS, and AB513134), were used to construct the phylogenetic tree. The group of transcontinental subtype is indicated by *yellow box*

The sequences were assembled according to the AB513134 sequence. The total number of reads was 244,723, and the average read length was 119.7 bp. The mean coverage was 2143 bp, and the alignments of all samples were satisfied with a minimum coverage of 20 bp. A consensus sequence for each sample was exported and analyzed for subtypes and mutations. First, we carried out a phylogenetic analysis using these whole genome sequences. Five complete HTLV-1 sequences, composed of two transcontinental subtypes (Rk13 and BOI) and three Japanese subtypes (ATK, ATL-YS and AB513134), were used to construct the phylogenetic tree. Of the 211 samples, 37 samples (18%) were classified as the transcontinental subtype and 174 (82%) were the Japanese subtype (Fig. 1). Among the three groups, the percentages of the transcontinental subtype in the f-HAM/TSP, s-HAM/TSP and AC groups were 33, 23 and 7%, respectively. The frequency of the transcontinental subtype was significantly higher in both f-HAM/TSP (χ^2^ test, *p* < 0.001) and s-HAM/TSP (χ^2^ test, *p* < 0.001) than in ACs (Table 1). The frequency of the transcontinental subtype was not different between f-HAM/TSP and ACs.

### HTLV-1 sequence changes in HAM/TSP and ACs

**Fig. 2:**
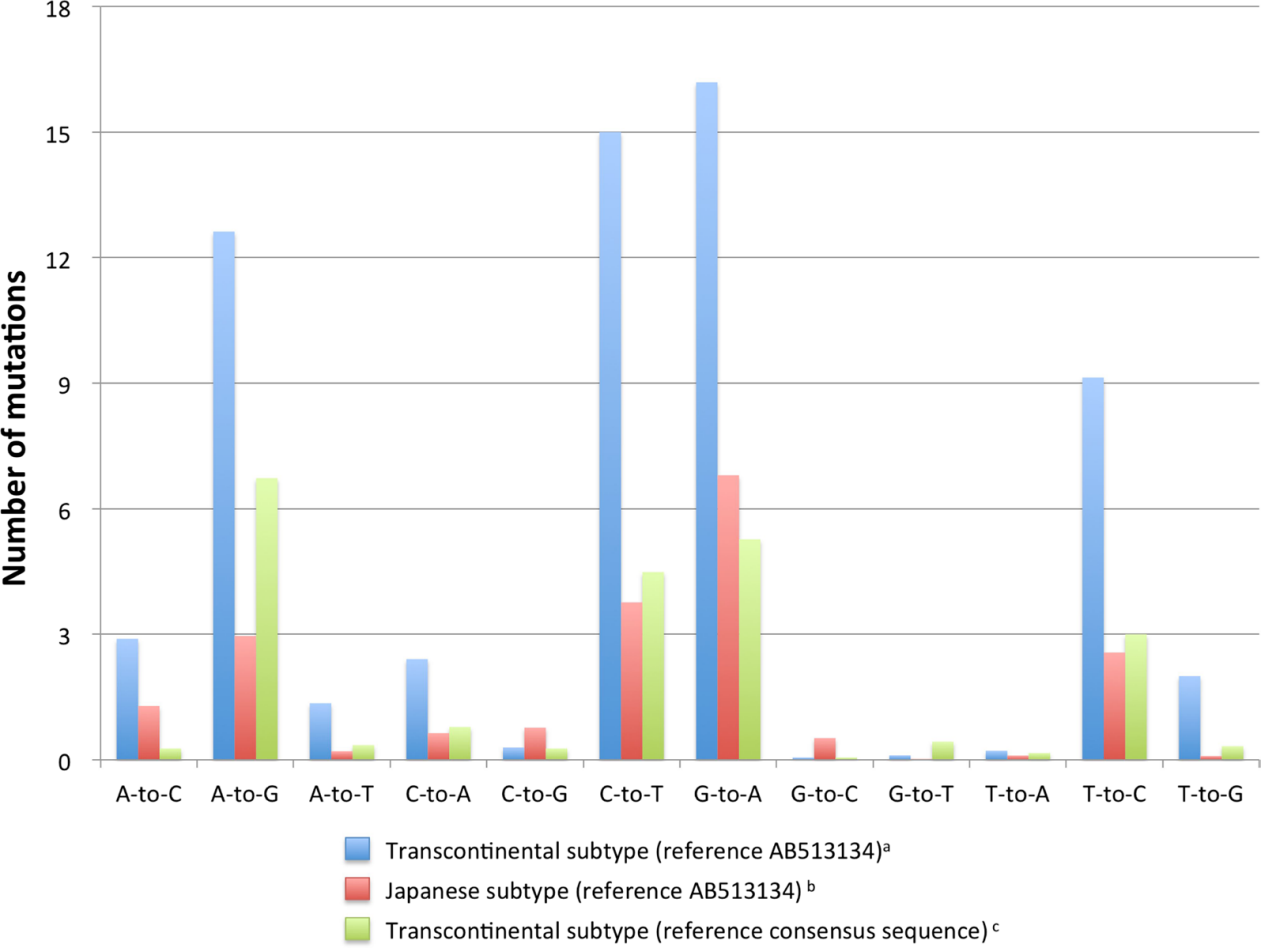
Frequency of mutations in the complete HTLV-1 sequences of 211 samples. (*Blue*) Mutations of transcontinental subtypes by mapping sequence to AB513134. (*Red*) Mutations of Japanese subtypes by mapping sequence to AB513134. (*Green*) Mutations of transcontinental subtypes by mapping sequence to consensus transcontinental reference

We compared the number of mutations among the groups, which were 34.2 in f-HAM/TSP, 29.6 in s-HAM/TSP and 22.3 in ACs on average. The number of mutations was significantly greater in both f-HAM/TSP and s-HAM/TSP patients than in ACs (ANOVA, *p* = 0.004) (Table 1). However, when comparing between two HTLV-1 subtypes, the number of mutations in the transcontinental subtype (62.3) was significantly higher than that in the Japanese subtype (19.7) (*t* test, *p* = 0.001). Therefore, we hypothesized that the larger number of mutations in HAM/TSP patients than ACs may be associated with the higher mutation rate in the transcontinental subtype in HAM/TSP patients rather than a difference in the clinical state. To confirm this assumption, we constructed the “transcontinental subtype reference” from the consensus sequences of the 37 transcontinental subtype samples analyzed in this study. We realigned the transcontinental subtype samples to the new consensus reference and analyzed the mutations. The mean number of mutations in the transcontinental subtype was 22.1, which was not different from the number of mutations in the Japanese subtype (22.3) mapped to AB513134. The number of mutations was also not different between HAM/TSP patients and ACs by analyzing each subtype. In terms of the type of nucleotide mutations, G-to-A mutations were the most frequent, a result similar to that of a previous report [18]. The G-to-A mutation was most frequent in the Japanese subtype, while in the transcontinental subtype, the A-to-G mutation was more common (Fig. 2). Next, we analyzed the nonsense mutations, insertions, and deletions in the HTLV-1 genome. To examine the mutations of each subtype, the sequences of Japanese subtype samples were mapped to AB513134, and the transcontinental subtype samples were mapped to “transcontinental subtype reference.” The frequencies of abortive genetic changes in the f-HAM/TSP, s-HAM/TSP, and AC groups were 6.7, 6.5 and 5.6%, respectively. There was no significant difference among these groups. The frequency in this study was lower than that of the ATL cases previously reported [18]. All mutations were nonsense mutations and no insertion or deletion mutations were detected (Table 2). G-to-A mutations were most frequent among the mutations causing nonsense mutations (10 of 14 mutations). This result was consistent with the previous report that G-to-A mutation was most frequent in ATL cases [18].

**Table 2:** Abortive genetic changes of HTLV-1 viral genes in HAM/TSP and ACs

Case	gag	pol	env	p12	p30	p13	p27	tax	HBZ
FHAM032									Q206*
FHAM033									Q206*
SHAM014				W87*					
SHAM027				W87*					
SHAM050				W82*					
SHAM065								R349*	
SHAM071				W87*					
SHAM092		W515*							
AC007					R208*	R54*			
AC009				W87*					
AC026		K725*							
AC065		K725*							
AC081				W87*					

Stop codons are indicated by an asterisk

*W* tryptophan, *K* lysine, *R* arginine, *Q* glutamine

### Comparison of HTLV-1 sequences between HAM/TSP patients and ACs

We analyzed complete sequences to determine whether HAM/TSP patients had specific mutations. To exclude bias, we chose one case in each of the paired familial cases, and a total of 113 HAM/TSP patients (21 f-HAM/TSP and 92 s-HAM/TSP) and 89 ACs were included. First, the sequences of all samples were mapped to AB513134, and fifty mutations were significantly more frequent in HAM/TSP patients than in ACs. However, all mutations that were significantly more frequent in HAM/TSP patients were common in the transcontinental subtype. Among these mutations, ten common mutations in the HTLV-1 provirus genes causing amino acid changes were specific to the transcontinental subtype (Table 3). We compared complete HTLV-1 sequences to clinical states but could not detect any relevant mutations in HAM/TSP.

**Table 3:** Ten common mutations causing amino acid changes detected in transcontinental subtype in the coding regions of HTLV-1

HTLV-1 proviral gene	AA change	Nucleotide variation
pol	Q323H	3963A > T
env	F19L	5237T > C
env	H137Q	5593C > A
p12	P23S	6903C > T
p30	L27R	6908T > G
p30	D140G	7247A > G
p30	A149T	7273G > A
tax	A221 V	7962C > T
tax	S304 N	8211G > A
HBZ	S13P	7247A > G

### Paired analysis of HTLV-1 sequences in familial HAM/TSP patients

To investigate whether de novo mutations occur in the major clones of HTLV-1 provirus among family members, we conducted paired analysis of HTLV-1 complete sequences in nine families. Among these families, two consisted of parent–child, six had siblings, and one had cousins. The virus sequence was scarcely different within pairs, except for one family (Table 4). The sequences were more different in Family 7 than in other families. This result could be because one member in Family 7 was affected with ATL in addition to HAM/TSP. We could conduct one paired analysis with HAM/TSP and the other with the HTLV-1 carrier in the same family; there was no sequence difference between them. The HTLV-1 provirus sequences showed few differences among family members, indicating that de novo mutations rarely occur. When developing ATL, proviral sequences may be changed. The clinical data of these family members is shown in Additional file 1.

**Table 4:** HTLV-1 provirus sequences in familial HAM/TSP by paired analysis

Family	Relationships	HTLV-1 subtypes	Number of variants within family
1	Parent–child	Transcontinental	0
2	Parent–child	Transcontinental	0
3	Siblings	Transcontinental	1
4	Siblings	Japanese	1
5	Siblings	Japanese	2
6	Siblings	Japanese	2
7^a^	Siblings	Japanese	35
8	Siblings	Japanese	1
9	Relatives	Japanese	3
10	Parent–child	Japanese	0

Both patients in Family 1–9 were developed with HAM/TSP, and in Family 10 one were affected with HAM/TSP and the other with HTLV-1 carrier

^a^One in Family 7 was affected with adult T-cell lymphoma in addition to HAM/TSP

### Host restriction factors in HAM/TSP

We tried to detect the specific mutations in HAM/TSP patients compared with ACs and identified many somatic mutations in HAM/TSP and ACs, however, we could not find significant differences between them. We examined the host restriction factors in HAM/TSP patients and HTLV-1 carriers. In HIV infection, APOBEC3D/F/G/H, TRIM5α, tetherin and SAMHD1 have been reported to be host restriction factors. Therefore, we selected these factors as candidate genes for development with HAM/TSP. We obtained polymorphisms and nonsynonymous or nonsense mutations of the genes using the whole exome sequence analysis. All target sequences were achieved with a minimum of 20× coverage. To eliminate bias, we chose one HAM/TSP patient in each family. Therefore, a total of 113 HAM/TSP patients and 89 ACs were included in this analysis. We detected two polymorphisms and four rare variants in APOBEC3G. The group with rare variants in APOBEC3G tended to have more G-to-A mutations than the group without rare variants (8.8 vs. 6.5, *p* = 0.054) (Table 5). Three polymorphisms and three rare variants were identified in TRIM5α. The patients with the rare variants were exclusively detected in the HAM/TSP group and tended to show higher PVLs than those without the rare variants (1088 vs. 596 copies/10^4^ PBMC, *p* = 0.151). The patients with the TRIM5α 136Q allele showed lower PVLs than those with 136R allele (354 vs. 654 copies/10^4^ PBMC, *p* = 0.003). The patients with the 304L variant of TRIM5α had significantly higher PVLs than those with 304H (1669 vs. 595 copies/10^4^ PBMC, *p* = 0.025) (Table 6). LD was detected between TRIM5α rs10838525 (136Q) and rs144528596 (304L) (r^2^ = 0.001, D′ = 1) (Additional file 2). The value of r^2^ was low, but D′ was high. Because allele frequency of 304L was rare, D′ estimates might inflate. There was no patient who had 136Q and 304L in common. We could not find any predominant mutations in these host restriction factors in HAM/TSP patients. No polymorphisms or mutations were detected in tetherin (Additional file 3).

**Table 5:** Rare variants and polymorphisms of host restriction factors identified in HAM/TSP and ACs

Gene	Analysis	Frequency			Number of G-to-A mutations of HTLV-1 sequence		
		HAM/TSP	ACs	*p*	Minor allele	Major allele	*p*
APOBEC3G	Rare variants	0.01	0.03	0.322	8.8 ± 4.1	6.5 ± 2.3	0.054
	All	0.22	0.26	0.538	6.8 ± 2.2	6.4 ± 2.4	0.309
TRIM5α	Rare variants	0.05	0.00	0.035	5.2 ± 1.7	6.6 ± 2.4	0.157
	All	0.49	0.47	0.834	6.4 ± 2.3	6.6 ± 2.4	0.614

Data are mean ± SD

**Table 6:** Characterization of TRM5α SNPs and proviral loads in HAM/TSP and ACs

AA change in TRIM5α	Minor allele frequency	HTLV-1 proviral loads (copies/10^4^ PBMC)		
		Minor allele	Major allele	*p*
H43Y	0.265	623 ± 894	606 ± 802	0.897
G110R	0.009	514 ± 344	612 ± 829	0.867
V112F	0.123	637 ± 790	608 ± 832	0.871
R136Q	0.137	354 ± 401	654 ± 870	0.003
H304L	0.014	1669 ± 1411	595 ± 809	0.025
F379 V	0.005	495	612 ± 828	NA

Data are mean ± SD

## Discussion

In this study, we demonstrated that the transcontinental subtype was significantly more frequent in HAM/TSP patients, especially in f-HAM/TSP patients, than in ACs. We constructed a phylogenic tree based on the complete sequence data and divided it into subtypes. There are two distinct subtypes in Japan; the most frequently observed (nearly 80%) HTLV-1 subtypes belong to the Japanese subtype, while the minor transcontinental subtype (20%) seems to cluster in the southern islands of Kyushu and Ryukyu [30]. Furukawa et al. [12] reported the association between transcontinental subtype and the risk of HAM/TSP. However, almost previous studies demonstrated that the nucleotide substitutions in some fragments of HTLV-1 genome are specific for the geographic origin of the patients rather than for the type of associated pathologies [31, 32]. In endemic area of HTLV-1, including the area examined in previous reports, one of the HTLV-1 subtypes was predominantly widespread. Kagoshima is a unique area where both HTLV-1 subgroups exist. Therefore, it is an appropriate region in which to evaluate whether the HTLV-1 subtype could influence disease development. Additionally, most previous reports except for a report by Furukawa et al. examined relatively few patients. We examined 211 samples and showed that the transcontinental subtype was more frequent in HAM/TSP patients, especially in familial cases, than in ACs. Comparison of the sequences of transcontinental subtype with those of Japanese subtype showed two transcontinental subtype-specific substitutions that changed amino acid in the *tax* gene [12]. A recent study showed similar results with 42 HAM/TSP patients and that the HBZ mRNA expression was significantly higher in HAM/TSP patients with Japanese subtype than in those with transcontinental subtype [33], while we analyzed the complete sequence of the transcontinental subtype in Japan and detected the transcontinental subtype-specific substitutions. Analyzing viral genes other than tax and HBZ between HAM/TSP patients and ACs might be useful for clarifying the differences in development with HAM/TSP between two HTLV-1 subtypes.

HTLV-1 has remarkably low genetic variability. In this study, the number of single nucleotide polymorphisms (SNPs) was relatively high in HAM/TSP patients compared to ACs when we analyzed all HTLV-1 sequences using the same reference of the Japanese subtype, which was the most prevalent in Japan. However, when we segregated the HTLV-1 subtypes and analyzed them using the references adequate for each subtype, the number of SNPs was not different, and the overall divergence was less than one percent. We could not find any specific mutations in HAM/TSP patients. It is unclear whether HAM/TSP-specific mutations could be detected by using a larger number of samples because the mutations in the HTLV-1 sequence are relatively rare. We examined a complete consensus sequence in this study, but it may be beneficial to analyze the quasispecies and integration sites of the HTLV-1 provirus. Recently, quasispecies-derived mechanisms have been shown to mediate the adaptability for persistence to escape from the host immune responses and/or to acquire resistance to antiviral agents, especially in HIV, hepatitis C virus (HCV) and hepatitis B virus (HBV) [34]. The frequency of mutations was small in the region with sufficient coverage in our study. The integration site has been studied in ATL. Hot spots of integration could not be identified, but the association of nearby host genes has been related to hematologic malignancies [35]. Identifying the integration site would also be useful in studying HAM/TSP. Furthermore, G-to-A mutations were common and caused the most nonsense mutations. These results suggested that APOBEC3G might exert an effect on viral mutations [18]. We examined the host APOCEC3G gene by whole exome sequencing and analyzed the association between polymorphisms and/or mutations in the host and the number of SNPs and/or G-to-A mutations in the HTLV-1 sequence, but no significant difference was detected. The frequencies of nonsense, insertion and deletion mutations of HTLV-1 viral genes in the HAM/TSP and AC groups were 6.6 and 5.6%, and which were fewer than previously reported for ATL cases (46.7%) [18]. The lower frequencies of insertion and deletion mutations could be due to MiSeq using short reads.

The examination of the HTLV-1 sequences intra-family, even among HTLV-1 carriers, has scarcely reported. Examination of the sequences of gp46 showed no difference between carriers and their partners experienced of seroconversion [36]. In this study, we found few differences within a family, despite examining complete sequences. Although there were only two parent–child pairs, pediatric cases had an earlier age of onset. We recently reported that the mean age of onset of 40 familial HAM/TSP cases was 41 years. Compared to this value, not only children but also parents had an earlier age of onset. Although we could not accurately identify the source of the infection, a carrier child whose parent developed HAM/TSP needs to be carefully followed up. A previous report for PVL between familial carriers showed variations. In this study, PVLs, anti-HTLV-1 antibody titer, and motor disability score were relatively similar in HAM/TSP cases.

We examined the host restriction factors known in HIV infection, such as APOBEC3, TRIM5α and SAMHD1, in HAM/TSP patients and carriers. APOBEC3 proteins are members of the cytidine deaminase family, which facilitates deamination during reverse transcription, resulting in nucleotide mutations. APOBEC3G deaminates cytosine residues in single-stranded DNA during reverse transcription, causing G-to-A hypermutations and inhibiting the viral replication. These editing enzymes likely evolved to inhibit retrovirus replication, including that of HIV [15, 16]. In HTLV-1, a nucleocapsid domain inhibits the incorporation of APOBEC3G into the virions and escape from the antiviral effect [37]. While this activity is not foolproof, APOBEC3G induce G-to-A mutations and consequently causes nonsense mutations [18]. An analysis of these restriction mechanisms has been conducted in vitro and in vivo; however, a population-based study has never been attempted to establish a relationship among host gene mutations, clinical states, and viral properties. This study is the first report of the host restriction factors in HTLV-1-associated diseases.

First, we examined APOBEC3G, and the patients having rare variants tended to show more G-to-A mutations than those without rare variants. However, no significance differences were observed in the clinical states, PVLs or viral mutations. Other APOBEC3 family members showed no significant difference. Next, we examined TRM5α, and patients having two polymorphisms in TRIM5α showed a significant difference in PVLs. TRIM5α is characterized by a RING finger zip binding domain, one or two B-box domains and a coiled-coil domain; it exhibits RING domain-dependent E3 ubiquitin ligase activity [38, 39]. TRIM5α recognizes the entry of retroviral capsids into the cytoplasm and blocks viral infection [40]. Several reports have described the relationship between TRIM5α genetic-variants and HIV disease progression. In this study, the 136Q polymorphism showed lower PVLs than 136R. The 136Q allele was more frequent in HIV-1 resistant individuals in US-based natural history and Pumwani cohort studies [41, 42]. Codon 136 is located within the coiled-coil domain. A switch from arginine to glutamine could be implicated in TRIM5α oligomerization and increased antiviral activity. The rare variant of 304L showed higher PVLs than 304H and was exclusively harbored in HAM/TSP patients. H304L is a noncoding variant located in the 3′UTR. The influence of this variant remains unknown and may be an accidental coincidence. Collectively, TRIM5α may influence host resistance to HTLV-1 infection.

It is important to examine the host-virus interaction to understand infectious disorders. One of the approaches to elucidate such interaction is to identify the polymorphisms and/or mutations in human and viral genomes. Emerging new technologies for nucleotide sequencing allow us to obtain vast numbers of sequencing data. It is difficult to detect the mutations associated with the disease and the pathophysiology in these massive data. Many new mutations were identified to combine the various analyzing methods in ATL [43]. Case–control studies such as genome-wide association studies, which require large numbers of cases and controls, are useful strategies by which to detect susceptible genes in common diseases. However, obtaining a large sample size of rare diseases, including HAM/TSP, and analyzing the causative genes is difficult. Optimal analyses such as advanced bioinformatics analyses need to be performed. Analyzing families with many affected individuals may be more powerful and fruitful in finding rare causal variants [44, 45]. Multiple rare variants in COQ2 have been reported to be associated with multiple system atrophy by analyzing familial clustering [46]. It is important for us to conduct the studies in different ethnic populations to obtain a larger sample size and to examine the cases focusing on familial clustering that suggest genetic influence.

## Conclusions

In conclusion, the transcontinental subtype is more frequent in patients with HAM/TSP, especially in familial cases. Ten common mutations with amino acid changes in the transcontinental subtype compared with Japanese subtypes were detected and implicated in the development of HAM/TSP. A R136Q polymorphism in TRIM5α results in lower PVLs. This result indicates that TIRM5α may be implicated in HTLV-1 infection.

## Additional files

**Additional file 1.** Clinical characteristics and HTLV-1 provirus sequences in familial HAM/TSP by paired analysis. OMDS; Osame’s motor disability scale. *Particle aggregation method.

**Additional file 2.** Haploview plot of linkage disequilibrium (r^2^) between six TRIM5α SNPs.

**Additional file 3.** Rare variants and polymorphisms detected in HAM/TSP and ACs. Data are mean ± SD.
