# Supplementary material for: Effects of host restriction factors and the HTLV-1 subtype on susceptibility to HTLV-1-associated myelopathy/tropical spastic paraparesis
Source: Retrovirology. 2017 Apr 19;14:26. doi: 10.1186/s12977-017-0350-9 (PMC5395872; doi:10.1186/s12977-017-0350-9)
Supplement: Supplementary file 1 — Additional file 1. Clinical characteristics and HTLV-1 provirus sequences in familial HAM/TSP by paired analysis. OMDS; Osame’s motor disability scale. *Particle aggregation method. [file 12977_2017_350_MOESM1_ESM.docx]

**Supplement 1. Clinical characteristics and HTLV-1 provirus sequences in familial HAM/TSP by paired analysis**

| Family | HTLV-1 subtypes | Sex | Age of onset | Duration  of illness | Disease progression | OMDS | Proviral loads  (Copies/10^4^ PBMCs) | Anti-HTLV-1 antibodies^*^ | Number of SNPs | Number of  G-to-A mutations | Number of variants  within Family |
| --- | --- | --- | --- | --- | --- | --- | --- | --- | --- | --- | --- |
| 1 | transcontinental | female | 28 | 35 | slow | 5 | 1044 | 1024 | 13 | 2 | 0 |
|  | transcontinental | female | 14 | 21 | slow | 3 | 683 | 16384 | 13 | 2 |  |
| 2 | transcontinental | female | 42 | 11 | slow | 5 | 327 | 256 | 20 | 5 | 0 |
|  | transcontinental | female | 15 | 8 | slow | 6 | 198 | 256 | 20 | 5 |  |
| 3 | transcontinental | female | 43 | 7 | slow | 2 | 1700 | 8192 | 14 | 2 | 1 |
|  | transcontinental | female | 34 | 10 | slow | 3 | 1636 | 2048 | 13 | 2 |  |
| 4 | Japanse | female | 45 | 8 | slow | 5 | 265 | 2048 | 22 | 6 | 1 |
|  | Japanse | male | 23 | 49 | slow | 5 | 211 | 8192 | 21 | 6 |  |
| 5 | Japanse | female | 45 | 10 | slow | 4 | 1204 | 32768 | 22 | 6 | 2 |
|  | Japanse | male | 58 | 1 | rapid | 4 | 249 | 32768 | 22 | 6 |  |
| 6 | Japanse | female | 30 | 20 | slow | 2 | 817 | 16384 | 16 | 8 | 2 |
|  | Japanse | female | 44 | 3 | slow | 2 | 1992 | 8192 | 16 | 7 |  |
| 7 | Japanse | female | 51 | 8 | slow | 4 | 2718 | 16384 | 29 | 9 | **35** |
|  | Japanse | female | 65 | 11 | slow | 10 | 535 | 16384 | 18 | 5 |  |
| 8 | Japanse | male | 27 | 37 | slow | 7 | 2785 | 16384 | 28 | 8 | 1 |
|  | Japanse | female | 62 | 2 | rapid | 3 | 1091 | 2048 | 29 | 9 |  |
| 9 | Japanse | female | 63 | 6 | slow | 5 | 1752 |  | 15 | 7 | 3 |
|  | Japanse | male | 57 | 1 | rapid | 3 | 767 | 1024 | 16 | 9 |  |
| 10 | Japanse | female | 36 | 20 | slow | 6 | 122 | 2048 | 20 | 8 | 0 |
|  | Japanse | female |  |  |  |  | 147 | 2048 | 20 | 8 |  |

OMDS; Osame’s motor disability scale.

*Particle Aggregation Method.
