# Supplementary figures and images for: Effects of host restriction factors and the HTLV-1 subtype on susceptibility to HTLV-1-associated myelopathy/tropical spastic paraparesis
Source: Retrovirology. 2017 Apr 19;14:26. doi: 10.1186/s12977-017-0350-9 (PMC5395872; doi:10.1186/s12977-017-0350-9)

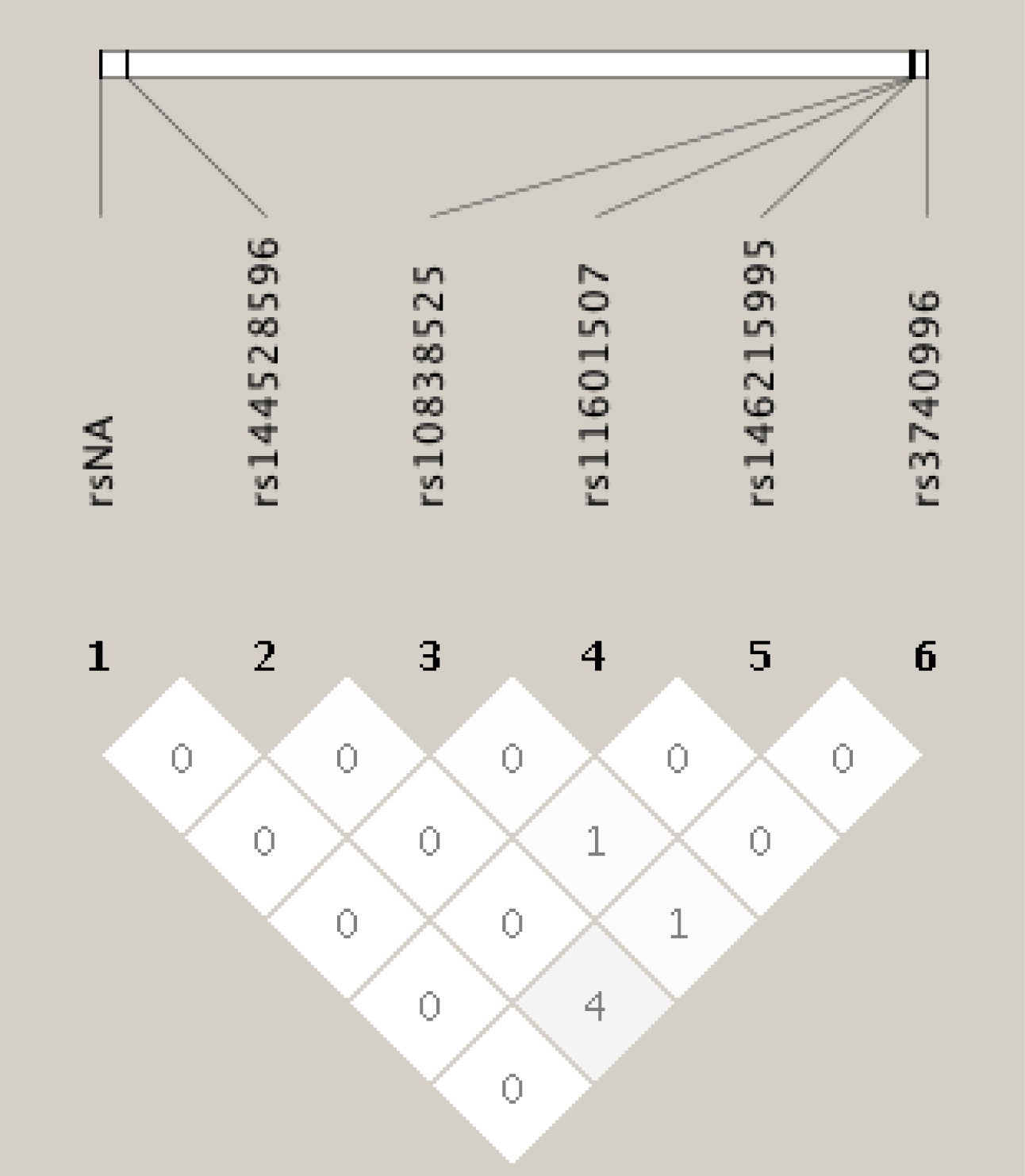

Supplement: Supplementary file 2 — Additional file 2. Haploview plot of linkage disequilibrium (r2) between six TRIM5α SNPs. [file 12977_2017_350_MOESM2_ESM.png]
