# Supplementary material for: Effects of host restriction factors and the HTLV-1 subtype on susceptibility to HTLV-1-associated myelopathy/tropical spastic paraparesis
Source: Retrovirology. 2017 Apr 19;14:26. doi: 10.1186/s12977-017-0350-9 (PMC5395872; doi:10.1186/s12977-017-0350-9)
Supplement: Supplementary file 3 — Additional file 3. Rare variants and polymorphisms detected in HAM/TSP and ACs. Data are mean ± SD. [file 12977_2017_350_MOESM3_ESM.docx]

**Supplement 3. Rare variants and polymorphisms detected in HAM/TSP and ACs**

|  |  |  |  |  |  |  |  |  |  |  | Proviral loads | | | Number of SNPs of HTLV-1 sequence | | | Number of G-to-A mutations of HTLV-1 sequence | | |
| --- | --- | --- | --- | --- | --- | --- | --- | --- | --- | --- | --- | --- | --- | --- | --- | --- | --- | --- | --- |
| Gene | Analysis | Chr | Position (hg19) | Amino acid change | 1000genome_  Frequency | HAM_Frequency | AC_Frequency | p | Minor Allele Frequency | Major Allele Frequency | Minor Allele | Major Allele | p | Minor Allele | Major Allele | p | Minor Allele | Major Allele | p |
| APOBEC3D |  | 22 | 39418864 | D19N |  | 0.01 | 0.00 |  | 0.005 | 0.953 | 1304 | 608±826 |  | 29 | 20.1±6.6 |  | 10 | 6.5±2.3 |  |
|  |  | 22 | 39418932 | I41M |  | 0.00 | 0.01 |  | 0.005 | 0.953 | 229 | 613±827 |  | 10 | 20.1±6.6 |  | 2 | 6.5±2.3 |  |
|  |  | 22 | 39427783 | E283K | 0.0018 | 0.00 | 0.02 |  | 0.009 | 0.948 | 160±64 | 616±828 | 0.438 | 13.5±5.0 | 20.2±6.6 | 0.155 | 4.5±0.7 | 6.5±2.4 | 0.226 |
|  |  | 22 | 39428247 | P363S |  | 0.00 | 0.01 |  | 0.005 | 0.953 | 306 | 613±827 |  | 23 | 20.1±6.6 |  | 8 | 6.5±2.4 |  |
|  | All |  |  |  |  | 0.01 | 0.04 | 0.172 | 0.024 | 0.934 | 432±492 | 616±833 | 0.624 | 17.8±8.3 | 20.2±6.6 | 0.428 | 5.8±3.2 | 6.5±2.4 | 0.496 |
| APOBEC3F |  | 22 | 39439066 | R48C | 0.0023 | 0.02 | 0.03 |  | 0.024 | 0.934 | 328±420 | 618±833 | 0.439 | 20.4±4.7 | 20.1±6.7 | 0.924 | 6.6±1.5 | 6.5±2.4 | 0.931 |
|  |  | 22 | 39441037 | T88I |  | 0.01 | 0.01 |  | 0.009 | 0.948 | 162±116 | 615±828 | 0.441 | 19.5±5.0 | 20.1±6.6 | 0.895 | 8.0±1.4 | 6.5±2.4 | 0.37 |
|  |  | 22 | 39441201 | R143C | 5.00E-04 | 0.00 | 0.01 |  | 0.005 | 0.953 | 114 | 613±827 |  | 17 | 20.1±6.6 |  | 5 | 6.5±2.4 |  |
|  |  | 22 | 39441517 | F179S |  | 0.01 | 0.02 |  | 0.014 | 0.943 | 124±106 | 618±830 | 0.304 | 20.3±3.8 | 20.1±6.6 | 0.955 | 7.7±1.2 | 6.5±2.4 | 0.393 |
|  | All |  |  |  |  | 0.03 | 0.07 | 0.186 | 0.043 | 0.915 | 236±320 | 628±838 | 0.164 | 20.0±4.0 | 20.1±6.7 | 0.956 | 6.8±1.5 | 6.5±2.4 | 0.728 |
| APOBEC3G |  | 22 | 39473374 | P3R |  | 0.01 | 0.00 |  | 0.005 | 0.953 | 272 | 613±827 |  | 49 | 20.0±6.3 |  | 14 | 6.5±2.3 |  |
|  |  | 22 | 39477566 | H186R | 0.12 | 0.17 | 0.17 |  | 0.161 | 0.796 | 528±596 | 628±865 | 0.523 | 20.9±4.8 | 20.0±6.9 | 0.479 | 6.9±1.9 | 6.4±2.4 | 0.353 |
|  |  | 22 | 39479867 | R238H | 5.00E-04 | 0.00 | 0.01 |  | 0.005 | 0.953 | 54 | 614±827 |  | 15 | 20.1±6.6 |  | 8 | 6.5±2.4 |  |
|  |  | 22 | 39482299 | G251S |  | 0.00 | 0.01 |  | 0.005 | 0.953 | 14 | 614±827 |  | 22 | 20.1±6.6 |  | 9 | 6.5±2.4 |  |
|  |  | 22 | 39482371 | Q275E | 0.06 | 0.04 | 0.07 |  | 0.052 | 0.905 | 287±368 | 630±841 | 0.181 | 18.6±5.2 | 20.2±6.7 | 0.418 | 6.2±2.1 | 6.5±2.4 | 0.636 |
|  |  | 22 | 39482527 | T327A |  | 0.00 | 0.01 |  | 0.005 | 0.953 | 394 | 612±828 |  | 10 | 20.2±6.6 |  | 4 | 6.5±2.4 |  |
|  | Rare Variants |  |  |  |  | 0.01 | 0.03 | 0.322 | 0.019 | 0.938 | 184±180 | 620±831 | 0.297 | 24.0±17.4 | 20.0±6.3 | 0.68 | 8.8±4.1 | 6.5±2.3 | **0.054** |
|  | All |  |  |  |  | 0.22 | 0.26 | 0.538 | 0.227 | 0.730 | 455±541 | 660±892 | 0.134 | 20.6±6.6 | 20.0±6.6 | 0.595 | 6.8±2.2 | 6.4±2.4 | 0.309 |
| APOBEC3H |  | 22 | 39496336 | R18L | 0.2 | 0.20 | 0.30 |  | 0.237 | 0.720 | 462±581 | 660±888 | 0.141 | 19.9±4.8 | 20.2±7.1 | 0.75 | 6.5±2.2 | 6.5±2.4 | 0.973 |
|  |  | 22 | 39496357 | P25R | 9.00E-04 | 0.00 | 0.02 |  | 0.009 | 0.948 | 399±528 | 613±829 | 0.716 | 21.0±1.4 | 20.1±6.6 | 0.85 | 8.0±2.8 | 6.5±2.4 | 0.37 |
|  |  | 22 | 39497300 | E70G | 0.0014 | 0.03 | 0.01 |  | 0.019 | 0.938 | 1260±1080 | 598±818 | 0.113 | 19.8±6.7 | 20.1±6.6 | 0.911 | 6.3±1.3 | 6.5±2.4 | 0.824 |
|  |  | 22 | 39497412 | F107L | 5.00E-04 | 0.01 | 0.00 |  | 0.005 | 0.953 | 2173 | 603±820 |  | 14 | 20.2±6.6 |  | 4 | 6.5±2.4 |  |
|  |  | 22 | 39498533 | A187V |  | 0.00 | 0.01 |  | 0.005 | 0.953 | 39 | 614±827 |  | 10 | 20.2±6.6 |  | 5 | 6.5±2.4 |  |
|  |  | 22 | 39498560 | C196Y |  | 0.01 | 0.01 |  | 0.009 | 0.948 | 97±119 | 616±828 | 0.378 | 17.5±2.1 | 20.2±6.6 | 0.575 | 5.5±3.5 | 6.5±2.4 | 0.543 |
|  | Rare Variants |  |  |  |  | 0.04 | 0.06 |  | 0.047 | 0.910 | 824±966 | 600±819 | 0.403 | 18.0±5.3 | 20.2±6.7 | 0.299 | 6.1±2.1 | 6.5±2.4 | 0.574 |
|  | All |  |  |  |  | 0.24 | 0.33 | 0.206 | 0.265 | 0.692 | 497±617 | 655±891 | 0.227 | 19.9±4.8 | 20.2±7.2 | 0.782 | 6.5±2.2 | 6.5±2.4 | 0.923 |
| TRIM5 |  | 11 | 5701281 | H43Y | 0.12 | 0.27 | 0.26 |  | 0.256 | 0.701 | 623±894 | 606±802 | 0.897 | 19.8±5.6 | 20.2±7.0 | 0.659 | 6.4±2.2 | 6.6±2.4 | 0.566 |
|  |  | 11 | 5701080 | G110R |  | 0.02 | 0.00 |  | 0.009 | 0.948 | 514±344 | 612±829 | 0.867 | 18.5±0.7 | 20.1±6.6 | 0.729 | 5.5±0.7 | 6.5±2.4 | 0.543 |
|  |  | 11 | 5701074 | V112F | 0.06 | 0.10 | 0.13 |  | 0.109 | 0.848 | 637±790 | 608±832 | 0.871 | 20.3±5.6 | 20.1±6.7 | 0.913 | 6.2±1.9 | 6.6±2.4 | 0.528 |
|  |  | 11 | 5701001 | R136Q | 0.21 | 0.12 | 0.18 |  | 0.137 | 0.820 | 354±401 | 654±870 | **0.003** | 20.3±5.7 | 20.1±6.8 | 0.843 | 6.8±2.6 | 6.5±2.3 | 0.434 |
|  |  | 11 | 5686870 | H304L | 0.01 | 0.03 | 0.00 |  | 0.014 | 0.943 | 1669±1411 | 595±809 | **0.025** | 16.7±2.5 | 20.2±6.6 | 0.363 | 4.7±2.5 | 6.5±2.3 | 0.173 |
|  |  | 11 | 5686386 | F379V |  | 0.01 | 0.00 |  | 0.005 | 0.953 | 495 | 612±828 |  | 23 | 20.1±6.6 |  | 6 | 6.5±2.4 |  |
|  | Rare Variants |  |  |  |  | 0.05 | 0.00 | **0.035** | 0.028 | 0.929 | 1088±1107 | 596±815 | 0.151 | 18.3±2.9 | 20.2±6.7 | 0.503 | 5.2±1.7 | 6.6±2.4 | 0.157 |
|  | All |  |  |  |  | 0.49 | 0.47 |  | 0.460 | 0.498 | 562±783 | 656±864 | 0.416 | 19.9±5.5 | 20.3±7.5 | 0.71 | 6.4±2.3 | 6.6±2.4 | 0.614 |
| SAMHD1 |  | 20 | 35575190 | A76S |  | 0.01 | 0.00 |  | 0.005 | 0.953 | 272 | 613±827 |  | 49 | 20.0±6.3 |  | 14 | 6.5±2.3 |  |

Data are mean ± SD.
